# Joint Modelling of Confounding Factors and Prominent Genetic Regulators Provides Increased Accuracy in Genetical Genomics Studies

**DOI:** 10.1371/journal.pcbi.1002330

**Published:** 2012-01-05

**Authors:** Nicoló Fusi, Oliver Stegle, Neil D. Lawrence

**Affiliations:** 1 Sheffield Institute for Translational Neuroscience, University of Sheffield, Sheffield, United Kingdom; 2 Machine Learning and Computational Biology Research Group, Max Planck Institute for Developmental Biology, Tübingen, Germany; University of Chicago, United States of America

## Abstract

Expression quantitative trait loci (eQTL) studies are an integral tool to investigate the genetic component of gene expression variation. A major challenge in the analysis of such studies are hidden confounding factors, such as unobserved covariates or unknown subtle environmental perturbations. These factors can induce a pronounced artifactual correlation structure in the expression profiles, which may create spurious false associations or mask real genetic association signals. Here, we report PANAMA (Probabilistic ANAlysis of genoMic dAta), a novel probabilistic model to account for confounding factors within an eQTL analysis. In contrast to previous methods, PANAMA learns hidden factors jointly with the effect of prominent genetic regulators. As a result, this new model can more accurately distinguish true genetic association signals from confounding variation. We applied our model and compared it to existing methods on different datasets and biological systems. PANAMA consistently performs better than alternative methods, and finds in particular substantially more *trans* regulators. Importantly, our approach not only identifies a greater number of associations, but also yields hits that are biologically more plausible and can be better reproduced between independent studies. A software implementation of PANAMA is freely available online at http://ml.sheffield.ac.uk/qtl/.

## Author Summary

The computational analysis of genetical genomics studies is challenged by confounding variation that is unrelated to the genetic factors of interest. Several approaches to account for these confounding factors have been proposed, greatly increasing the sensitivity in recovering direct genetic (*cis*) associations between variable genetic loci and the expression levels of individual genes. Crucially, these existing techniques largely rely on the true association signals being orthogonal to the confounding variation. Here, we show that when studying indirect (*trans*) genetic effects, for example from master regulators, their association signals can overlap with confounding factors estimated using existing methods. This technical overlap can lead to overcorrection, erroneously explaining away true associations as confounders. To address these shortcomings, we propose PANAMA, a model that jointly learns hidden factors while accounting for the effect of selected genetic regulators. In applications to several studies, PANAMA is more accurate than existing methods in recovering the hidden confounding factors. As a result, we find an increase in the statistical power for direct (*cis*) and indirect (*trans*) associations. Most strikingly on yeast, PANAMA not only finds additional associations but also identifies master regulators that can be better reproduced between independent studies.

## Introduction

Genome-wide analysis of the regulatory role of polymorphic loci on gene expression has been carried out in a range of different study designs and biological systems. For example, association mapping in human has uncovered an abundance of *cis* associations that contribute to the variation of a third of all human genes [1], [2]. In segregating yeast strains, linkage studies have revealed extensive genetic *trans* regulation, with a few regulatory hotspots controlling the expression profiles of tens or hundreds of genes [3], [4].

Despite the success of such expression quantitative trait loci (eQTL) studies, it has also become clear that the analysis of these data comes along with non-trivial statistical hurdles [5]. Different types of external confounding factors, including environment or technical influences, can substantially alter the outcome of an eQTL scan. Unobserved confounders can both obscure true association signals and create new spurious associations that are false [6], [7].

Suitable data preprocessing, or careful design of randomized studies are helpful measures to avoid confounders in the first place [8], however they rarely rule out confounding influences entirely. It is also relatively straightforward to account for those factors that are known and measured. For example, it is standard procedure to include covariates such as age and gender in the analysis [9], [10]. Similarly, the effect of populational relatedness between samples, a confounding effect that is observed or can be reliably estimated form the genotype data [11], [12], is usually included in the model. However other factors, including subtle environmental or technical influences, often remain unknown to the experimenter, but still need to be accounted for. Their potential impact has previously been characterized in multiple studies; for example Plagnol et al. [13] and Locke et al. [14] showed that virtually any aspect of sample handling can impact the analysis.

Several computational methods have been developed to account for unknown confounding variation within eQTL analyses [2], [6], [7], [15], [16]. A common assumption these methods built on is that confounders are prone to exhibit broad effects, influencing large fractions of the measured gene expression levels. This characteristic has been exploited to learn the profile of hidden confounders using models that are related to PCA [2], [6], [15]. Once learnt, these factors can then be included in the analysis analogously to known covariates. Another branch of methods avoids recovering the hidden factors explicitly, instead correcting for the correlation structure they induce between the samples [7], [16]. Here, the inter-sample correlation is estimated from the expression profiles first, to then account for its influence in an association scan using mixed linear models. Both types of methods have been applied in a number of studies. Advantages versus naive analysis include better-calibrated test statistics [16] and improved reproducibility of hits between independent studies [7]. Perhaps most strikingly, statistical methods to correct for hidden confounders have also been shown to substantially increase the power to detect eQTLs, increasing the number of significant *cis* associations by up to 3-fold [2], [17].

While improved sensitivity to detect *cis*-acting eQTLs is an important and necessary step, we expect that even more valuable insights can be gained from those loci that regulate multiple target genes in *trans*. The interest in these regulatory hotspots has been tremendous in recent years, but limited reproducibility between studies has been a concern (see for example the discussion in Breitling et al. [18]). Accurate correction for confounding factors is key to improve the reliability of these regulatory associations, however statistical overlap between confounding factors and true association signals from downstream effects can hamper the identification and fitting of confounders. For example, methodology that merely accounts for broad variance components, such as PCA, is doomed to fail. If the effect size of *trans* regulatory hotspots is large enough, they induce a correlation structure that is similar to the one caused by confounding factors. As a result, true *trans* regulators tend to be mistaken for confounders and are erroneously explained away.

Here, we report an integrated probabilistic model PANAMA (Probabilistic ANAlysis of genoMic dAta) to address these shortcoming of established approaches. PANAMA learns a dictionary of confounding factors from the observed expression profiles. Unique to PANAMA is to jointly learn these factors while accounting for the effect of loci with a pronounced *trans* regulatory effect, thereby avoiding overlaps between true genetic association signals and the covariance structure induced by the learnt confounders. The statistical model underlying our algorithm is simple and computationally tractable for large eQTL datasets. PANAMA is based on the framework of mixed linear models, and combines the advantages of factor-based methods, such as PCA, SVA [6] or PEER [2], [15] with methods that estimate the implicit covariance structure induced by confounding variation [12], [16]. The model is fully automated and can be easily adapted to include additional observed confounding sources of variation, such as population structure or known covariates.

We applied PANAMA to a range of eQTL studies, including synthetic data and studies from yeast, mouse and human. Across datasets, PANAMA performed better than previous methods, identifying a greater number of significant eQTLs and in particular additional *trans* regulators. We provide multiple sources of evidence that the associations recovered by PANAMA are indeed likely to be real. Most strikingly in yeast, the findings by PANAMA can be better reproduced between independent studies and are more consistent with prior knowledge about the underlying regulatory network. Finally, we also give insights into the limitations of current methods to account for confounders that help to understand the relationship between confounding variation, *cis* regulation and *trans* effects.

## Results

### Learning of confounding factors in the presence of *trans* regulators

**Figure 1:**
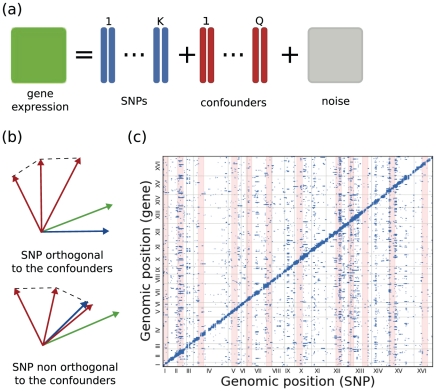
Illustration of the PANAMA model. (**a**) Representation of the linear model used by PANAMA to correct for the effect of confounding factors. (**b**) Alternative settings of confounders in relation to true genetic signals: First, orthogonality between confounders and genetics. The variation in the gene expression levels (green arrow) can be better explained by the SNP (blue arrow). Second, statistical overlap between variation explained by confounders and the genetic variation as often found in *trans* hotspots. Gene expression variation can be equally well explained as genetic or due to a confounding factor. Previous methods focus in the first setting, while PANAMA is able to handle both situations. (**c**) PANAMA applied to the yeast eQTL dataset. Pronounced *trans* regulators that overlap with the learnt confounding factors are highlighted in red.

The statistical model underlying PANAMA assumes additive contributions from true genetic effects and hidden confounding factors. Briefly, this linear model expresses the gene expression of gene 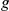 measured in 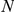 individuals as the sum of weighted contributions from a set of 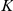 SNPs 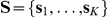 as well as 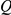 confounders 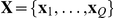, a mean term 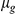 and a noise term 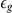 (See Figure 1a)
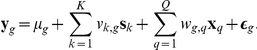
Neither the regression weights 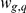 nor the profiles of the confounding factors 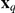 are known *a priori* and hence need to be learnt from the expression data. Parameter inference in PANAMA is done in the mixed model framework [12], [19]. In this hierarchical model, the regression weights of the hidden factors are marginalized out, yielding a covariance structure in a multivariate Gaussian model to capture the effect of confounders. Intuitively, the objective during learning in PANAMA is to explain the empirical correlation structure between samples shared across genes by the state of the hidden factors. In the presence of extensive *trans* regulation this approach leads to over-correction, running the risk of explaining away true genetic association signals. To circumvent this side effect, PANAMA also includes a subset of all SNPs in the model, resulting in a more complete covariance structure that satisfies an appropriate balance between explaining confounding variation and preserving true genetic signals (Figure 1b,c). In this approach, the variance contribution of few major signal SNPs and the state of the hidden factors are then jointly estimated. Moreover, an appropriate number of hidden factors is determined automatically during learning. As a result, PANAMA is statistically robust and inference of hidden factors is feasible without manual setting of any tuning parameters. Additional observed covariates, if available, can also be included in the model; see Methods and the supplementary Text S1 for full details.

### Simulation study

The evaluation of methods to call eQTLs is difficult as reliable ground truth information is not available. Following previous work [2], [20], [21], we have used synthetic data to assess and compare PANAMA with alternative approaches. To minimize assumptions we need to impose on the simulation procedure we created an artificial dataset that borrows key characteristics from a real eQTL study in yeast [4] (See also Application to segregating yeast strains). In this approach, we first fit PANAMA to the original yeast eQTL data, thereby estimating the number of *cis* and *trans* associations, an empirical distribution of effect sizes, and finally the characteristics of confounding variation. Based on these estimates we recreated an in silico eQTL dataset using standard linear assumptions; see Text S1 for full details on the exact approach. To rule out possible biases of this dataset towards our method, we additionally considered a simulation setting when fitting the ICE model [7] to the real data for estimating simulation parameters (see below).

**Figure 2:**
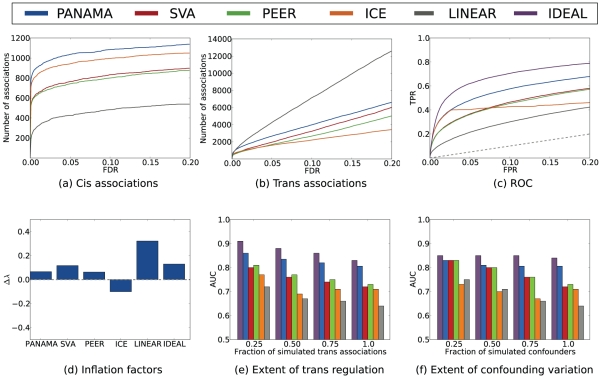
Evaluation of PANAMA and alternative methods on the simulated eQTL dataset. (**a,b**) number of recovered *cis* and *trans* associations as a function of the chosen false discovery rate cutoff. To circumvent biases due to linkage, at most one association per chromosome and gene is counted. (**c**) Receiver Operating Characteristics (ROC) for recovering true simulated associations, depicting the true positive rate (TPR) as a function of the permitted false positive rate (FPR). (**d**) inflation factors, defined as 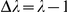, indicating either inflated p-value distributions (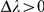) or deflation (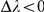) of the respective tests statistics. (**e**) Area under the ROC curve for alternative simulated datasets, subsampling certain fractions of number of simulated *trans* association. (**f**) Area under the ROC curve for alternative simulated datasets, subsampling the number of simulated confounding factors.

Given the synthetic eQTL study, we employed alternative methods to recover the underlying simulated associations. We compared PANAMA to standard linear regression (LINEAR), ignoring the presence of confounders entirely, as well as SVA [6], ICE [7] and PEER [2], [15], established and widely used approaches to correct for hidden confounders. For reference, we also compared to an idealized model with the simulated confounders perfectly removed (IDEAL). First, Figure 2a and 2b show the respective number of significant *cis* and *trans* associations as a function of the false discovery rate (FDR) cutoff. To avoid overly optimistic association counts due to linkage disequilibrium, we considered at most a single *cis* association per gene and at most one *trans* association per chromosome for each gene. PANAMA found more *cis* associations than any other approach and retrieved the greatest number of *trans* associations among methods that correct for hidden confounders. Notably, the linear model appeared to find even more *trans* associations, however the majority of these calls were inconsistent with the simulated ground truth and were spurious false positives. The extent of false associations called by the linear model is also reflected in Figure 2c, which shows the receiver operating characteristics for each method. All approaches that correct for confounders performed strikingly better than the linear model. Among these, PANAMA was most accurate, achieving greater sensitivity than any other method for a large range of false positive rates (FPR), approaching the performance of an ideal model (IDEAL).

Since some models, including SVA and PEER, allow to account for additional known covariates, we investigated their performance when adding the strongest genetic regulators as covariates. This procedure is mimicking the central concept of PANAMA using previous methods. However, comparative results (Supplementary Figure S7) show that iterative learning of PANAMA still performs significantly better.

Next, we studied the statistics of obtained p-values, checking for departure from a uniform distribution that either indicates inflation (genomic control 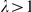) or deflation (genomic control 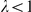) of the respective methods (Figure 2d and Supplementary Figure S8 for corresponding Q-Q-plots). All methods except for ICE yielded an inflated p-value distribution. Notably, this observation also applies to the ideal model where the effect of confounders had been perfectly removed. Thus, in settings with sufficiently strong *trans* regulation, inflated statistics are not necessarily due to poor calibration because of confounders, but instead may occur as a consequence of an excess of true biological signals themselves. We also checked that calls by the various methods were not overly optimistic and artificially inflated. Indeed, false discovery rate estimates from all methods but the linear model were approximately in line with the empirical rate of errors when taking the ground truth into account (Supporting Figure S1), with PANAMA being the best calibrated method.

We then repeated the same analysis on a broader range of simulated datasets, varying particular aspects of the simulation procedure around the parameters obtained from the fit to the real yeast data. Figure 2e shows the accuracy of alternative methods when reducing the extent of simulated *trans* regulation by subsampling from the set of initial *trans* effects. These results highlight that previous methods only work well in the regime of little *trans* regulation, while PANAMA provides for accurate calls for a wider range of settings. Similarly, Figure 2f shows results for strong *trans* regulation, now varying the extent of confounding factors from weaker to stronger influences. Again, PANAMA was found to be more robust than previous approaches, recovering true simulated associations with great accuracy irrespectively of the magnitude of simulated confounding.

Finally, we investigated the impact of the exact of model used to fit the association characteristics to the initial yeast dataset. Supporting Figure S2 shows summary results for a second synthetic dataset fitted using ICE. As ICE tends to be the most conservative approach among the considered methods, the extent of *trans* regulation on this simulated data was severely reduced. As a consequence, the differences between methods were considerably smaller, however confirming the previously observed trends.

### Application to segregating yeast strains

Having established the accuracy of PANAMA in recovering hidden confounders, we applied PANAMA and the alternative methods to the primary eQTL dataset from segregating yeast strains [4]. These data cover a set of 108 genetically diverse strains that have been expression profiled in two environmental conditions, glucose and ethanol. First, we focused on the glucose condition, which has previously been expression profiled [3], providing an independent study for the purpose of comparison.

**Figure 3:**
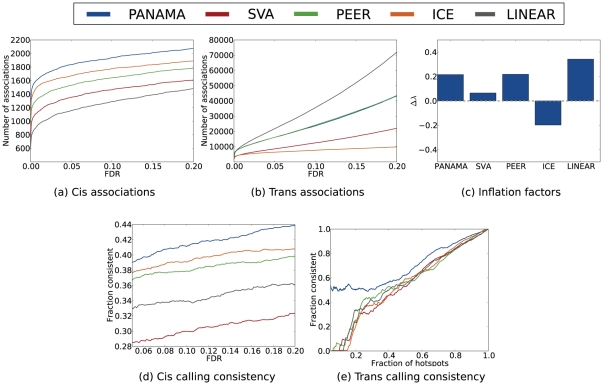
Evaluation of alternative methods on the eQTL dataset from segregating yeast strains (glucose condition). (**a,b**): number of *cis* and *trans* associations found by alternative methods as a function of the chosen FDR cutoff. (**c**) Inflation factors of alternative methods, defined as 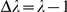. (**d**) Consistency of calling *cis* associations between two independent glucose yeast eQTL datasets. (**e**) Consistency of calling eQTL hotspots between two independent glucose yeast datasets, where SNPs are ordered by the extent of *trans* regulation as determined by 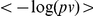.

Figure 3a and 3b show the number of *cis* and *trans* associations for different methods as a function of the FDR cutoff. Again, we considered at most one association per chromosome to avoid confounding the size of associations with their number. In line with previously reported results [2], [7] and the simulated setting (Simulation study), the standard linear model identified fewer *cis* associations than methods that correct for confounding variation. The trends from the simulated dataset also carried over for *trans* associations, where the linear model called many more associations than methods that account for confounders, yielding an excess of regulatory hotspots (See Supporting Figure S3). It has previously been suggested that many of these are likely to be false; see for example the discussion in Kang et al. [7]. Among the methods that correct for confounding variation, PANAMA identified the greatest number of associations. Among the alternative methods, ICE appeared to be more sensitive in recovering *cis* associations while PEER and SVA retrieved a greater number of *trans* associations. Also note that models that account for confounding factors yielded slightly inflated p-value distributions (Figure 3c, Supplementary Figure S9), supporting that also in real settings, a certain degree of inflation may be caused by extensive *trans* regulation. Finally, supporting Figure S3 shows the number of associations called by different methods as a function of the genomic position. This summary of genome-wide eQTLs confirms that ICE is most conservative in detecting hotspots, whereas all other methods do find multiple *trans* bands. For comparison we also included a version of PANAMA that also corrects for the *trans* regulators that are accounted for while learning (PANAMA_trans_ , see Methods and supporting Text S1). PANAMA_trans_ yields near-identical results to ICE, which explains the differences and similarities between the two approaches, where PANAMA can be regarded as generalization of ICE. By accounting for pronounced regulators PANAMA circumvents the over-conservative correction of the ICE model.

#### Reproducibility of eQTLs between studies

To objectively shed light on the validity of the associations called, we considered the consistency of calls between two independent studies. The glucose environment from Smith et al. [4] has previously been studied [3], sharing a common set of segregants. We checked the consistency in calling genes with a *cis* association for increasing FDR cutoffs (Figure 3d). Alternatively, focusing on the consistency of regulatory hotspots, Figure 3e shows the ranking consistency of polymorphisms ordered by their regulatory potential on multiple genes. Reassuringly, for both *cis* effects and *trans* regulatory hotspots, PANAMA yielded results with far greater consistency than any other currently available method. In particular the consistency of *trans* hotspots suggest that PANAMA achieved an appropriate balance between explaining away spurious signals as confounding variation and identifying hotspots that are likely to have a true genetic underpinning.

#### Consistency of *trans* regulatory hotspots with respect to known regulatory mechanisms in yeast

As a second means of validating *trans* eQTLs, we investigated to what extent polymorphisms that regulate multiple genes in *trans* can be interpreted as indirect effects that are mediated by known transcriptional regulators. For this analysis we considered an established regulatory network of transcription factors extracted from Yeastract [22]. Although we do not expect *trans* associations to be exclusively mediated by direct transcriptional regulation, the degree of associations that are consistent with this regulatory structure is nevertheless an informative indicator for the validity of eQTL calls from different models.

For each transcription factor, we considered polymorphisms in the vicinity of the coding region of the transcription factor (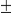
10 kb around the coding region), and tested the fraction of associations with genes that are known targets of the transcription factor versus other associations with genes that are no direct targets. Table S1 shows the F-score (harmonic mean between precision and recall) for each of 129 transcription factors that had at least one SNP in the local *cis* window. For half of the 129 TFs, PANAMA yielded a higher F-score than any of the other methods considered. Interestingly, the standard linear models performed second best under this metric, achieving the greatest F-score in 36% of all cases, followed by PEER (28%), SVA (15%) and ICE (6%). Among the methods that correct for confounders, PANAMA consistently yielded the highest F-score.

#### Detecting eQTLs that are shared across environments

Finally, we considered the full expression dataset from Smith et al. [4], combining expression measurement in an ethanol and glucose background. Because each yeast strain was profiled twice, the set of samples was not independent, but instead had a replicate population structure. Similarly as done in previous work [16], we accounted for this genetic relatedness in PANAMA by adding a population covariance term (Material and Methods). Supporting Figure S4 shows the number of associations retrieved by PANAMA and alternative methods on this joint yeast dataset. Because PANAMA accounted for the replicate structure of the dataset, the increase in the number of associations compared to the analysis of the single-condition analysis was modest. Other methods, not accounting for the replicate structure of the genotypes, yielded severely inflated test statistics, identifying a *trans* effect for the great majority of all genes. To check the impact of the population structure covariance, we also applied PANAMA without the correction for artificial genetic relatedness, yielding similarly inflated results (data not shown).

The complete set of eQTL calls from PANAMA, on the glucose condition alone and the joint analysis on both conditions, are available as Supporting Dataset S1 and Supporting Dataset S2 respectively.

### Application to further eQTL studies

We successfully applied PANAMA to additional ongoing and retrospective studies. For example, on a dataset from inbred mouse crosses [23], PANAMA identified a greater number of associations than other methods (Supplementary Figure S5). In contrast to the yeast dataset, the distribution of p-values on this dataset was almost uniform, suggesting that the extent of true *trans* regulation is lower. We also investigated parts of a dataset of the genetics of human cortical gene expression [24]. On chromosome 17, methods that account for confounders identified more genes in associations than a linear model, with SVA and PANAMA retrieving the greatest number (see supporting Figure S6). Results on other four other chromosomes were similar (data not shown).

Finally, results of PANAMA applied to an RNA-Seq eQTL study on *Arabidopsis* [25] indicate that expression heterogeneity as accounted for by PANAMA is also present on expression estimates from short read technologies, which is consistent with previous reports in human RNA-Seq studies [26]. This suggests that statistical challenges due to confounding variation are not specific to a particular platform for measuring gene expression.

## Discussion

We have reported the development of PANAMA, an advanced statistical model to correct for confounding influences while preserving genuine genetic association signals. We have shown that this approach is of substantial practical use in a range of real settings and studies. The correction approach of PANAMA, for the first time, is able to not only find more *cis* eQTLs, but also greatly improves the statistical power to uncover true *trans* regulators. PANAMA finds a greater number of associations, and calls eQTLs that are more likely to be real, as validated by means of realistic simulated settings and an analysis of eQTL consistency between independent studies. Most notably, PANAMA identified several strong *trans* hotspots on yeast, out of which at least 40% could be reproduced on a replication dataset.

There are several previous approaches to correct for confounding influences in eQTL studies. These methods can be broadly grouped into factor-based models like PCA, SVA [6] and PEER [2], [15], and approaches that employ a mixed linear model [7], [16], estimating a covariance structure that captures the confounding variation. An important reason why PANAMA performs well is the intermediate approach taken here, that is, learning a covariance structure within a linear mixed model (LMM), but at the same time retaining the low-rank constraint which yields an explicit representation of factors. Moreover, PANAMA systematically exploits the flexibility provided by the representation in terms of covariance structures, jointly accounting for genetic regulators while estimating the confounding factors. Our approach is stable and robust, avoiding the need to first subtract off the genetic contribution greedily, as for example suggested and implemented in SVA [6] and PEER [2], [15]. Although this is not the focus of this work, we have shown how our approach can be combined with additional measures to correct for observed sources of confounding variation, such as known covariates or populational relatedness. The utility of such measures has been illustrated in the joint analysis on data from two environmental conditions. A more specialized approach that is aimed at the combined correction for expression confounders and population structure has recently been proposed by Listgarten et al. [16]. This LMM-EH approach is methodologically related to what is done here, as the contribution from multiple sources of variation are combined within a single covariance structure. Importantly, the main contribution in PANAMA is an integrated model that does not include additional confounders but true genetic regulators. Unique to PANAMA, these regulators are jointly identified and accounted for during learning of the confounding factors. Our analysis shows, that this approach yields a significant improvement in the sensitivity of recovering *trans* associations and plausible regulatory hotspots. A tabular overview of the relation between alternative methods is shown in Supporting Table S2.

In conclusion, PANAMA is an important step towards exhaustively addressing common types of confounding variation in eQTL studies. The number of datasets that benefit from careful dissection of true genetic signals and confounders, as done here, is expected to rise quickly. Growing sample sizes and expression profiling in more than one environment allow for the estimation of more subtle confounding influences and at the same time provide the statistical power to detect many more *trans* effects than possible as of today.

## Materials and Methods

PANAMA is based on a linear additive linear model, accounting for effects from 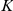 observed SNPs 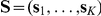 and contributions from a dictionary of 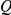 hidden factors 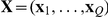. The resulting generative model for 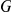 gene expression levels 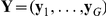 can then be cast as
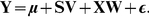
(1)We assume that expression levels and SNPs are observed in each of 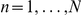 individuals, 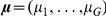 is a vector of gene-specific mean terms and ɛ denotes Gaussian distributed observation noise, 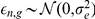. The matrices 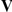 and 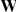 represent the weights for the SNP effects and hidden factor effects respectively. To improve the parameters estimation, we introduce a hierarchy on the weights of genetic influences and hidden factors in Equation (1). We marginalize out the effect of the latent factors, 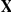 and a subset of the SNPs with a strong regulatory role (see below), resulting in a mixed linear model. We choose independent Gaussian priors for the factors weights 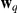 and the weights of respective SNPs 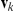


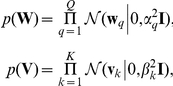
and integrate them out. The corresponding marginal likelihood, conditioned on the state of the confounding factors 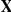 is now factorized across genes
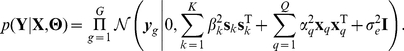
(2)For notational convenience we dropped the mean term 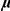 and we have defined 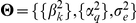, the set of all hyperparameters of the model.

### Known covariates

If available, additional covariates can directly be included in the background covariance structure from Equation (2)
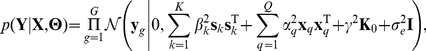
(3)where 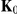 denotes the covariance induced by these additional covariates and 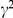 the corresponding scaling parameter. Examples for possible choices of this covariance include the covariance induced by a fixed covariate vectors, i.e. 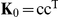 or a kinship matrix that accounts for the genetic relatedness (see for example Kang et al. [12] and Listgarten et al. [16]).

### Model fitting

The most probable state of the latent variables 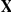 and the hyperparameters 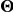 can be identified via a straightforward maximum likelihood approach
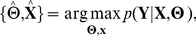
(4)for example employing a gradient-based optimizer. In practical applications of PANAMA, this model fitting (Equation (4)) is not carried out with the set of all genome-wide SNPs included in Equation (1), because the number of weight parameters 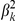 for each SNP would be prohibitive. Only those genetic regulators with strong effects on multiple genes do play a role during the estimation of hidden factors and thus need to be accounted for. Our inference scheme determines the set of relevant regulators in an iterative procedure. The number of hidden factors to be learnt, 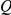 is not set *a priori* and instead 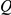 is set to a sufficiently large value. During the optimization, the individual variance parameters for each factors, 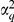, automatically determine an appropriate number of effective factors, switching off unused ones. For full details of the algorithm and analysis of the robustness of this approach see Supporting Text S1.

### Significance testing

Once the confounding-correcting covariance structure is determined from the maximum likelihood solution of Equation (4), significance testing can be carried out in the framework of mixed linear models. The association between a SNP 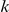 and gene 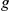 to be tested is treated as fixed effect, allowing to construct a likelihood ratio statistics of the form
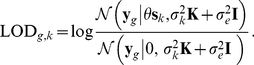
(5)Here, the covariance matrix 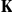 denotes the covariance structure explaining confounding variation, which is derived from the fitted PANAMA model. Computationally, the likelihood ratio tests (Equation (5)) can be efficiently implemented using recently proposed computational tricks [19], allowing for application to large-scale genomic data (Supporting Text S1).

In PANAMA, this correction covariance structure 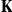 only accounts for the confounding factors, excluding the genetic regulators (See Equation (2))
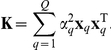
In PANAMA_trans_, also correcting for the *trans* factors, the covariance equals to
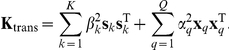
For computational efficiency we fix the covariance structure 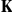 that is learnt from the full expression dataset upfront. The relative weighting of the covariance (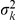) and the noise term (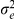) are then adjusted on the background and null model (Equation (5)) for every single test carried out, using recent advances for efficient mixed model inference [19].

### Yeast datasets

We used the yeast expression dataset from Smith et al. [4] (GEO accession number GSE9376), which consists of 5,493 probes measured in 109 segregants derived from a cross between BY and RM. The authors provided the genotypes, which consisted of 2,956 genotyped loci.

An association was defined as *cis* if the location of the SNP and the location of the opening reading frame (ORF) of the gene were within 10 kb, and *trans* otherwise. In order to validate the associations found, we also used data from Brem et al. [3] (GEO accession number GSE1990), which consisted of 7,084 probes and 2,956 genotyped loci in 112 segregants. For the purpose of comparison, we defined *cis* associations in the same way as we did for the previous dataset.

### Mouse dataset

We used the data described in Schadt [23], consisting of 23,698 expression measurements and 137 genotyped loci for 111 F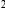 mouse lines.

### Human dataset

We used the dataset from [24] (GEO accession number GSE8919), which consists of 14,078 transcripts and 366,140 SNPs genotyped on 193 human samples.

### Yeastract

We used data from Yeastract [22], which contains information about the regulatory network between 185 transcription factors and 6,298 genes. Out of these 189 transcription factors, we selected the 129 TFs that had a polymorphism in the vicinity (10 kb) of the coding region.

## Supporting Information

Dataset S1 List of eQTL calls from PANAMA, on the glucose condition alone in the yeast dataset.
(CSV)

Dataset S2 List of eQTL calls from PANAMA in the joint analysis of both conditions (ethanol, glucose) in the yeast dataset.
(CSV)

Figure S1 Comparison of the calibration accuracy of false discovery estimates for alternative methods. Shown is the estimated false discovery rate (E(FDR)) as a function of the empirical false discovery rate for associations called on the simulated dataset. In summary, PANAMA is better calibrated than any other method, neither underestimating nor overestimating the FDR.
(PDF)

Figure S2 Receiver operating characteristics for an alternative simulated dataset based on a fit of ICE to the original yeast dataset. While the general performance differences are smaller, the general trends remain. The kink in ICE is due to deflation of the model. See the main paper Figure 2 for complementary results on a dataset simulated from PANAMA.
(PDF)

Figure S3 Number of associations called as a function of the genomic position for alternative methods on the eQTL dataset from segregating yeast strains (glucose condition).
(PDF)

Figure S4Evaluation of alternative methods on the eQTL dataset from segregating yeast strains (glucose and ethanol jointly). (**a,b**) number of recovered *cis* and *trans* associations as a function of the false discovery rate cutoff. At most one association per chromosome and gene was counted. (**b**) inflation factors, defined as 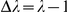. Note that PANAMA included a covariance term that accounts for the genetic relatedness of identical individuals profiled in two conditions. As a result, PANAMA yielded better calibrated results, calling fewer associations than other methods.(PDF)

Figure S5Evaluation of alternative methods on the eQTL dataset from mouse. (**a**) Number of *cis* and *trans* associations found by alternative methods as a function of the FDR cutoff. (**b**) Inflation factors of alternative methods, defined as 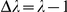.(PDF)

Figure S6 Number of associations as a function of the false discovery rate cutoff on the human dataset.
(PDF)

Figure S7 Receiver operating characteristics (ROC) curve comparing PANAMA to a modified version of SVA that models the most prominent genetic regulators as covariates.
(PDF)

Figure S8 Comparison of theoretical PV statistics with empirical distribution. Figure shows the quantile-quantile plots for alternative methods evaluated on the simulated dataset.
(PDF)

Figure S9 Comparison of theoretical PV statistics with empirical distribution. Figure shows the quantile-quantile plots for alternative methods evaluated on the yeast dataset.
(PDF)

Table S1 Comparison of the different models that account for confounders (SVA,PEER, ICE, LMM-EH, PANAMA) and LINEAR. A mark indicates that the model exhibits that property. The properties are: *Low rank*: is the model using a low-rank representation of the confounders? *LMM*: is it a linear mixed model? *Preserve genetic signal*: is the model explicitly preserving the genetic signal or is it greedily subtracting the confounding effects? PANAMA is the only model that spans all the different properties, since it imposes a low-rank structure for the confounders, but is efficiently implemented as a linear mixed model. Moreover, the latent confounders are learned in conjunction with the genetics, thereby preserving true genetic signals.
(PDF)

Table S2F-score (
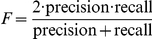
) for alternative methods in recovering known regulatory mechanisms from Yeastract.(PDF)

Text S1 Supplementary methods.
(PDF)
