# Supplementary figures and images for: Joint Modelling of Confounding Factors and Prominent Genetic Regulators Provides Increased Accuracy in Genetical Genomics Studies
Source: PLoS Comput Biol. 2012 Jan 5;8(1):e1002330. doi: 10.1371/journal.pcbi.1002330 (PMC3252274; doi:10.1371/journal.pcbi.1002330)

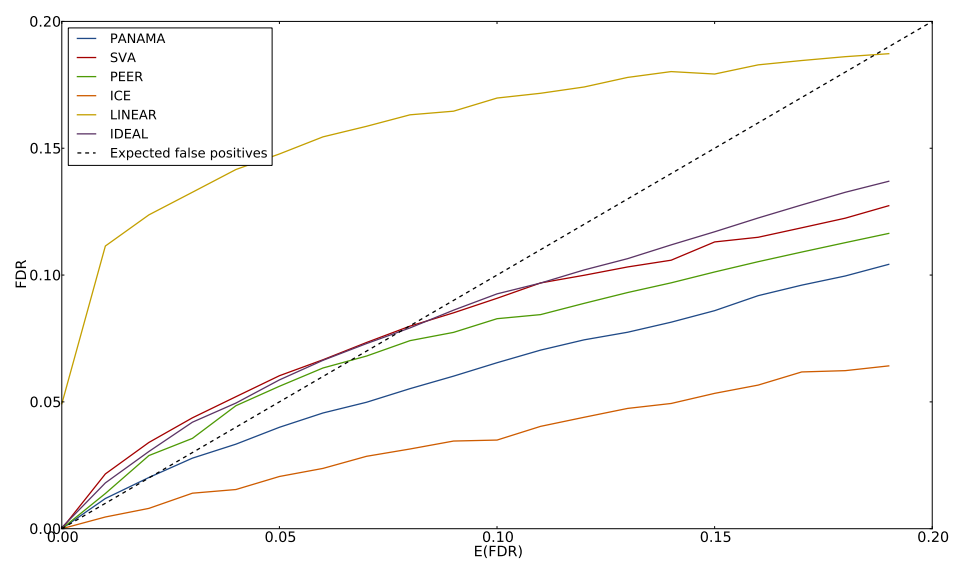

Supplement: Figure S1 — Comparison of the calibration accuracy of false discovery estimates for alternative methods. Shown is the estimated false discovery rate (E(FDR)) as a function of the empirical false discovery rate for associations called on the simulated dataset. In summary, PANAMA is better calibrated than any other method, neither underestimating nor overestimating the FDR. (PDF) [file pcbi.1002330.s003.pdf]

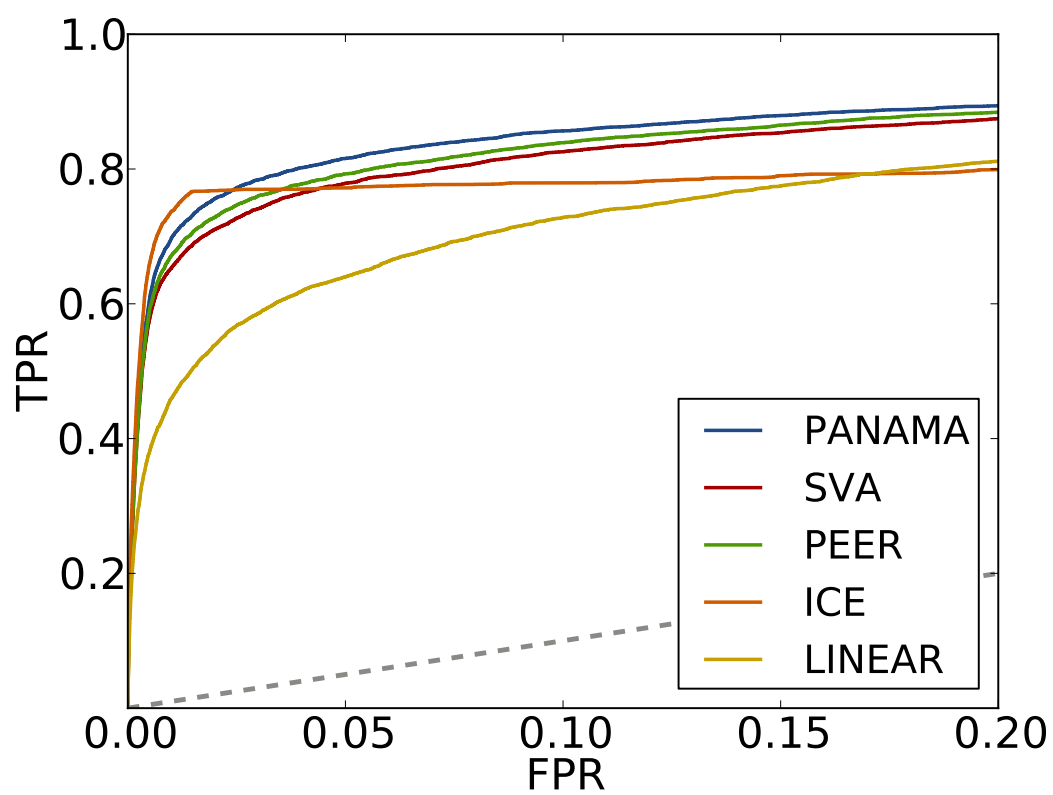

Supplement: Figure S2 — Receiver operating characteristics for an alternative simulated dataset based on a fit of ICE to the original yeast dataset. While the general performance differences are smaller, the general trends remain. The kink in ICE is due to deflation of the model. See the main paper Figure 2 for complementary results on a dataset simulated from PANAMA. (PDF) [file pcbi.1002330.s004.pdf]

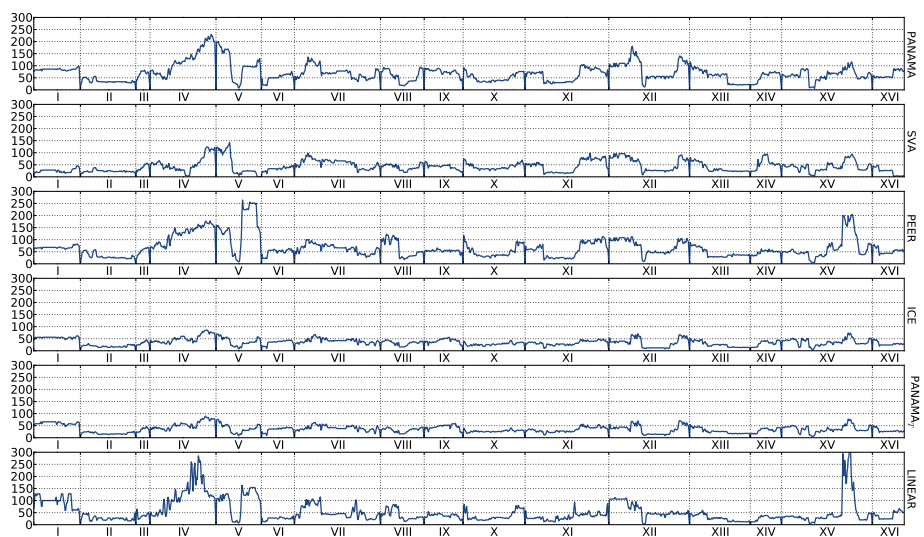

Supplement: Figure S3 — Number of associations called as a function of the genomic position for alternative methods on the eQTL dataset from segregating yeast strains (glucose condition). (PDF) [file pcbi.1002330.s005.pdf]

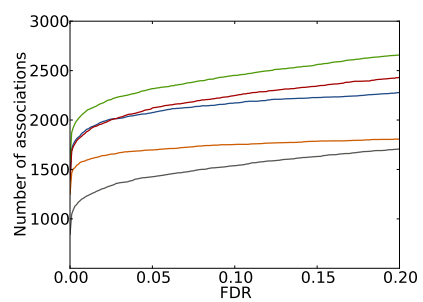

(a) Cis associations

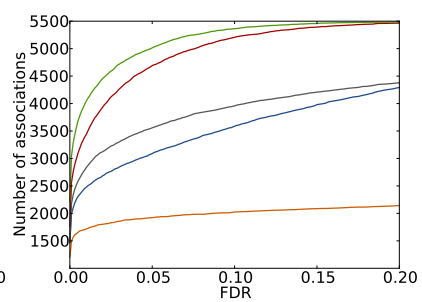

(b) Trans associations

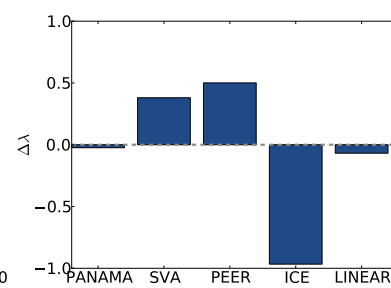

(c) Inflation factors

Supplement: Figure S4 — Evaluation of alternative methods on the eQTL dataset from segregating yeast strains (glucose and ethanol jointly). (a,b) number of recovered cis and trans associations as a function of the false discovery rate cutoff. At most one association per chromosome and gene was counted. (b) inflation factors, defined as . Note that PANAMA included a covariance term that accounts for the genetic relatedness of identical individuals profiled in two conditions. As a result, PANAMA yielded better calibrated results, calling fewer associations than other methods. (PDF) [file pcbi.1002330.s006.pdf]

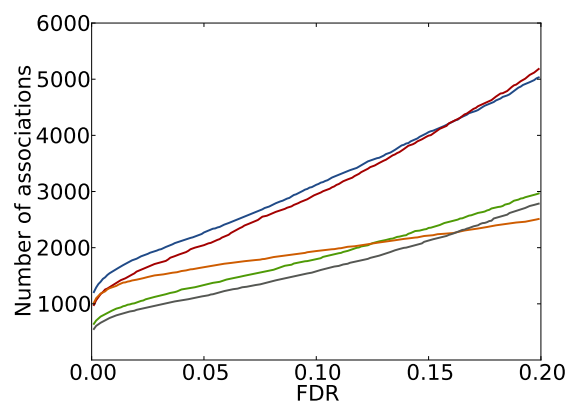

(a) Associations

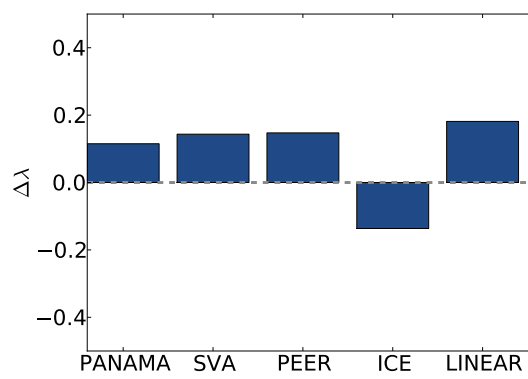

(b) Inflation factors

Supplement: Figure S5 — Evaluation of alternative methods on the eQTL dataset from mouse. (a) Number of cis and trans associations found by alternative methods as a function of the FDR cutoff. (b) Inflation factors of alternative methods, defined as . (PDF) [file pcbi.1002330.s007.pdf]

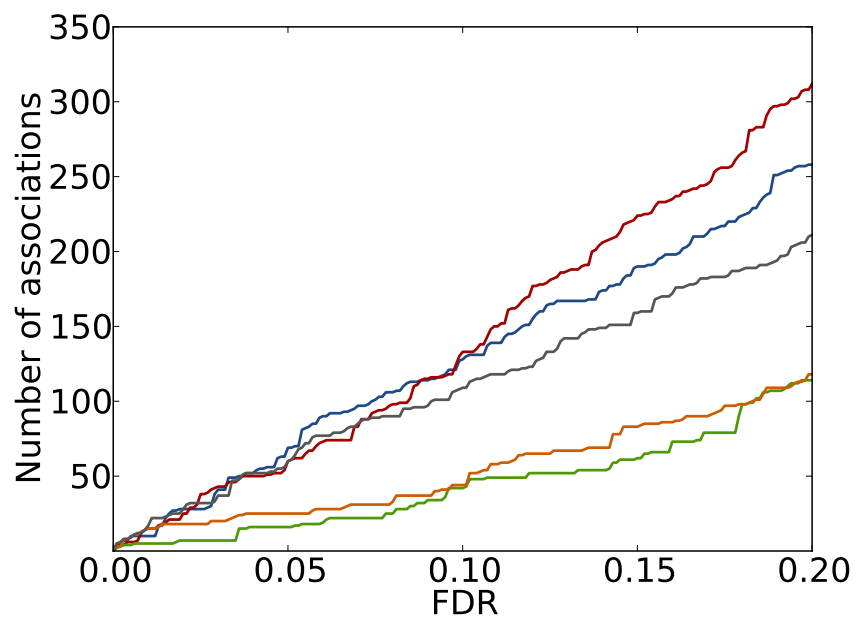

Supplement: Figure S6 — Number of associations as a function of the false discovery rate cutoff on the human dataset. (PDF) [file pcbi.1002330.s008.pdf]

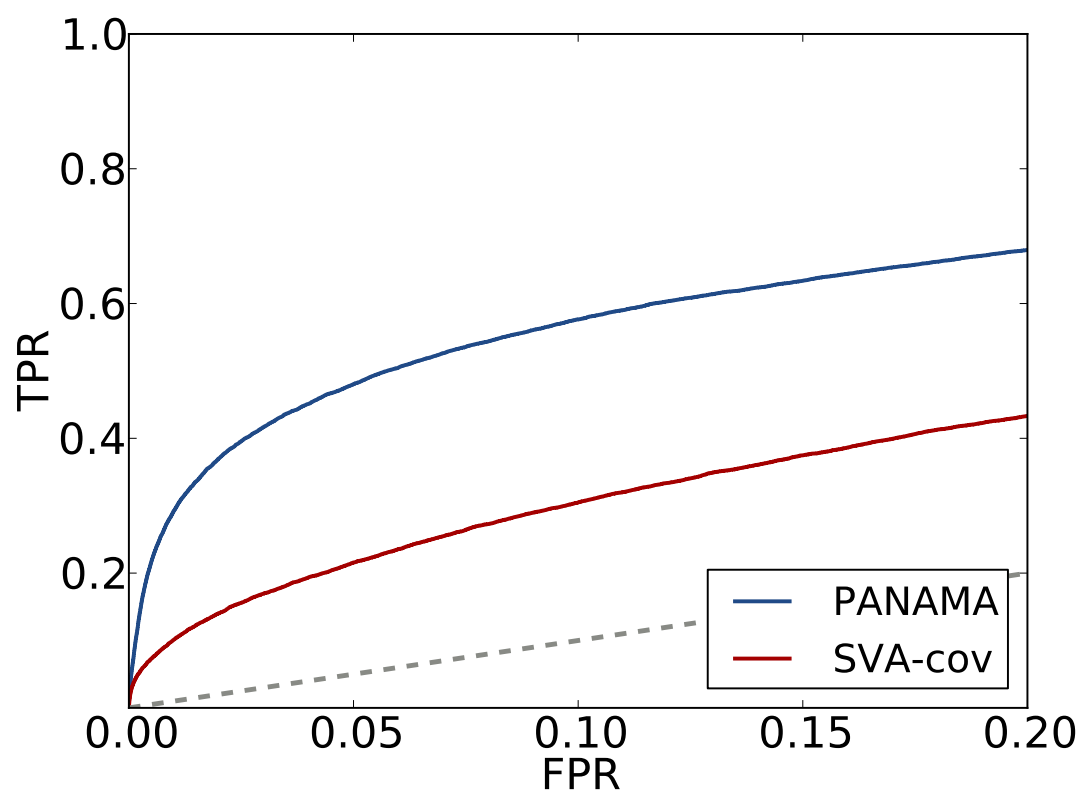

Supplement: Figure S7 — Receiver operating characteristics (ROC) curve comparing PANAMA to a modified version of SVA that models the most prominent genetic regulators as covariates. (PDF) [file pcbi.1002330.s009.pdf]

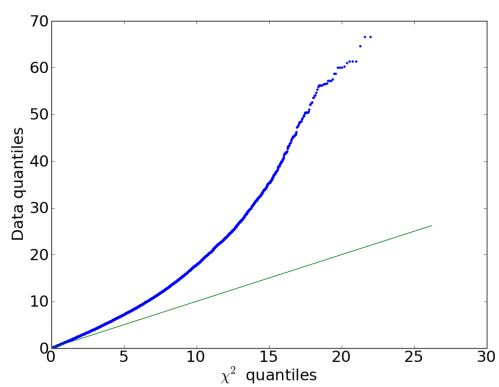

(a) Linear model

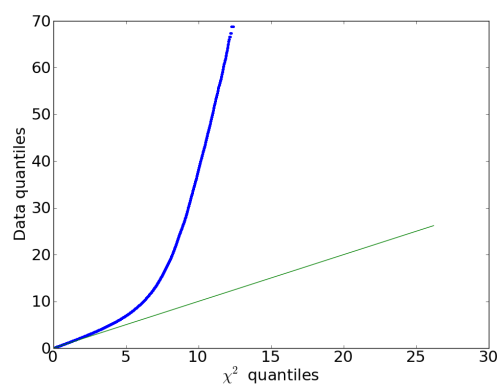

(b) Ideal model

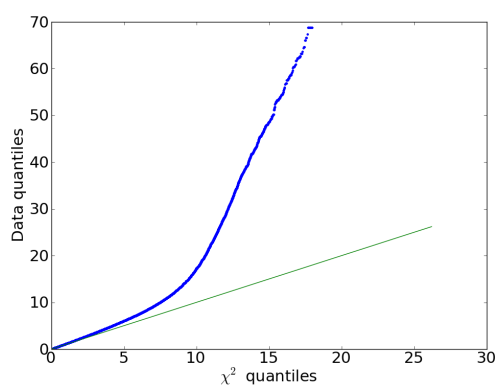

(c) SVA

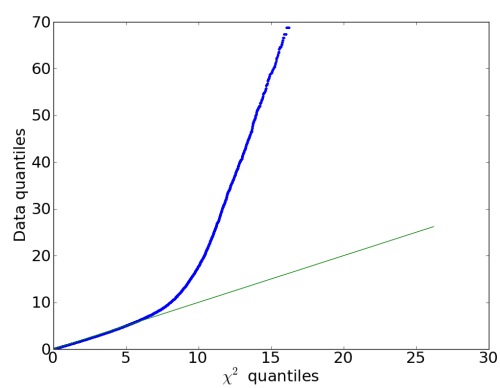

(d) ICE

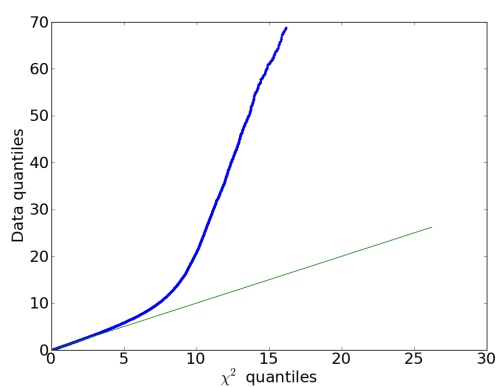

(e) PANAMA

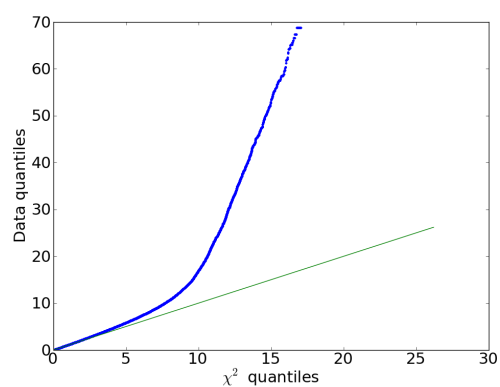

(f) PEER

Supplement: Figure S8 — Comparison of theoretical PV statistics with empirical distribution. Figure shows the quantile-quantile plots for alternative methods evaluated on the simulated dataset. (PDF) [file pcbi.1002330.s010.pdf]

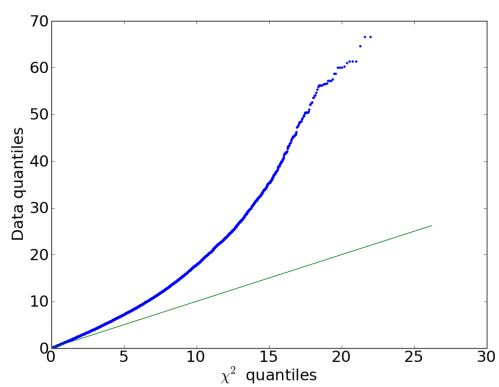

(a) Linear model

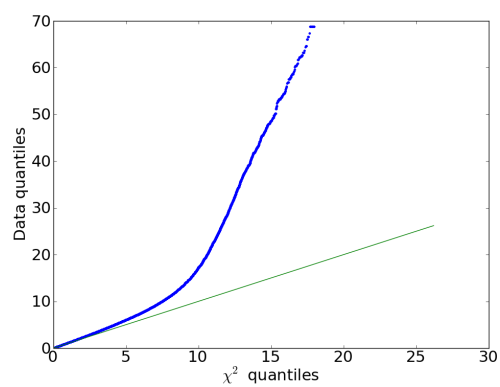

(b) SVA

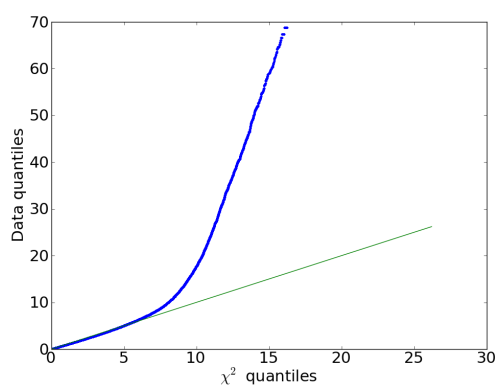

(c) ICE

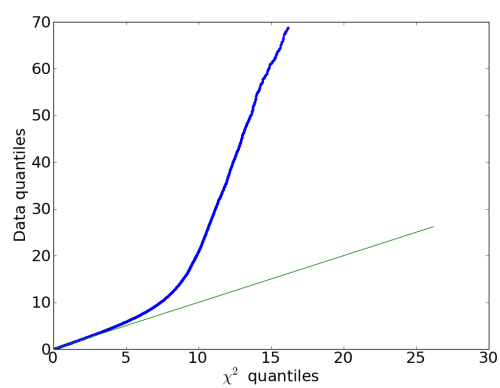

(d) PANAMA

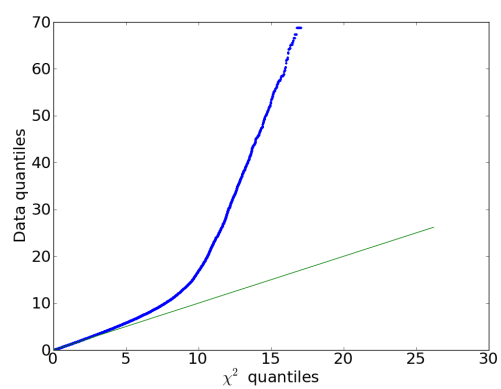

(e) PEER

Supplement: Figure S9 — Comparison of theoretical PV statistics with empirical distribution. Figure shows the quantile-quantile plots for alternative methods evaluated on the yeast dataset. (PDF) [file pcbi.1002330.s011.pdf]
