# Supplementary material for: Joint Modelling of Confounding Factors and Prominent Genetic Regulators Provides Increased Accuracy in Genetical Genomics Studies
Source: PLoS Comput Biol. 2012 Jan 5;8(1):e1002330. doi: 10.1371/journal.pcbi.1002330 (PMC3252274; doi:10.1371/journal.pcbi.1002330)
Supplement: Table S1 — Comparison of the different models that account for confounders (SVA,PEER, ICE, LMM-EH, PANAMA) and LINEAR. A mark indicates that the model exhibits that property. The properties are: Low rank: is the model using a low-rank representation of the confounders? LMM: is it a linear mixed model? Preserve genetic signal: is the model explicitly preserving the genetic signal or is it greedily subtracting the confounding effects? PANAMA is the only model that spans all the different properties, since it imposes a low-rank structure for the confounders, but is efficiently implemented as a linear mixed model. Moreover, the latent confounders are learned in conjunction with the genetics, thereby preserving true genetic signals. (PDF) [file pcbi.1002330.s012.pdf]

TF NAME	PANAMA	LINEAR	SVA	ICE	PEER
YLR228C	0.013	**0.026**	0.000	0.000	0.013
YOR372C	0.011	**0.044**	0.000	0.000	0.011
YMR042W	**0.044**	**0.044**	0.022	0.000	0.022
YIR023W	**0.014**	0.005	0.005	0.005	**0.014**
YHR124W	0.036	**0.059**	0.019	0.019	0.031
YOL028C	0.000	**0.201**	0.000	0.000	0.000
YOR172W	**0.133**	0.000	0.000	0.000	0.000
YNL103W	**0.022**	0.008	0.008	0.008	**0.022**
YDR213W	**0.010**	**0.010**	**0.010**	**0.010**	**0.010**
YHR206W	0.000	**0.902**	0.000	0.000	0.000
YDR034C	**0.020**	0.000	0.000	0.000	0.000
YMR280C	**0.031**	**0.031**	**0.031**	**0.031**	**0.031**
YML007W	0.020	**0.043**	0.011	0.008	0.015
YDR451C	0.019	**0.028**	0.009	0.005	0.019
YLR223C	0.033	**0.055**	0.000	0.022	0.033
YGL209W	**0.023**	0.000	0.000	0.000	0.000
YOR363C	0.071	**0.137**	0.000	0.000	0.024
YLR131C	0.071	**0.138**	0.000	0.000	0.071
YBR297W	0.059	0.059	**0.087**	0.030	0.059
YDL170W	0.042	**0.096**	0.000	0.000	0.042
YKL020C	**0.095**	**0.095**	0.000	0.000	**0.095**
YLR014C	0.000	**0.033**	0.000	0.000	0.000
YOR028C	**0.046**	0.028	0.035	0.028	**0.046**
YKL043W	**0.057**	0.019	0.019	0.019	0.019
YCR106W	**0.037**	0.028	0.009	0.000	0.019
YOR077W	0.022	**0.222**	0.011	0.011	0.011
YDR259C	**0.091**	**0.091**	0.047	0.069	0.047
YGL013C	**0.018**	0.012	0.012	0.012	**0.018**
YGL252C	**0.024**	**0.024**	0.000	0.000	**0.024**
YMR070W	**0.045**	0.023	**0.045**	**0.045**	**0.045**
YNL204C	**0.046**	0.013	0.013	0.000	0.013
YFL021W	0.054	**0.105**	0.027	0.027	0.054
YHR178W	**0.061**	0.000	0.000	0.000	0.000
YML027W	**0.031**	0.009	0.013	0.013	0.018
YGR288W	**0.016**	0.000	0.000	0.000	0.000
YJR060W	**0.060**	**0.060**	**0.060**	0.045	**0.060**
YKR099W	**0.020**	0.000	0.000	0.000	**0.020**
YBR066C	**0.022**	**0.022**	**0.022**	0.007	0.014
YML076C	**0.048**	0.024	0.010	0.005	0.039
YIR018W	0.042	**0.082**	0.042	0.042	0.042
YER028C	**0.016**	0.012	0.012	0.012	0.012
YER045C	**0.051**	0.026	0.039	0.026	0.039
YOL067C	0.079	**0.246**	0.027	0.040	0.079
YMR312W	0.075	**0.143**	0.000	0.019	0.075
YBL008W	**0.040**	0.012	0.028	0.004	0.028
YGR044C	**0.053**	0.000	0.000	0.027	**0.053**
YGL071W	0.039	**0.067**	0.000	0.000	0.029
YJL103C	**0.024**	**0.024**	0.000	0.000	0.000
YOL116W	**0.023**	0.000	0.000	0.000	0.000
YBL103C	0.037	**0.060**	0.000	0.000	0.037

TF NAME	PANAMA	LINEAR	SVA	ICE	PEER
YBR182C	**0.013**	0.009	0.009	0.004	**0.013**
YNL255C	**0.075**	0.000	0.038	0.038	0.038
YDL020C	**0.108**	0.056	0.056	0.056	0.056
YOR113W	**0.070**	0.036	**0.070**	0.036	**0.070**
YPL230W	**0.051**	0.000	0.000	0.000	0.026
YPR008W	**0.016**	0.000	0.000	0.000	**0.016**
YIL036W	**0.038**	0.033	0.017	0.017	**0.038**
YMR172W	**0.065**	0.049	0.033	0.033	**0.065**
YKL005C	**0.043**	**0.043**	**0.043**	0.000	**0.043**
YMR043W	**0.018**	0.008	0.008	0.005	0.005
YBR215W	0.070	**0.147**	0.000	0.043	0.070
YMR164C	**0.011**	0.000	0.000	0.000	0.000
YNL309W	0.074	**0.108**	0.006	0.011	0.074
YDR480W	**0.010**	0.000	**0.010**	0.000	**0.010**
YDR423C	**0.084**	0.043	0.043	0.064	**0.084**
YML051W	**0.017**	0.010	0.010	0.009	0.013
YFR034C	0.061	**0.091**	0.062	0.031	0.061
YKL185W	**0.052**	0.021	0.032	0.016	0.032
YDR253C	**0.040**	0.020	0.025	0.010	0.025
YOR380W	0.030	**0.073**	0.000	0.015	0.030
YHR006W	0.011	**0.033**	0.011	0.000	0.011
YER109C	**0.069**	0.018	0.035	0.009	0.018
YNL167C	**0.037**	0.022	**0.037**	0.029	**0.037**
YOL089C	0.072	**0.281**	0.000	0.012	0.072
YGL166W	**0.066**	**0.066**	**0.066**	0.000	**0.066**
YPL133C	0.009	**0.018**	0.000	0.000	0.000
YJL110C	**0.117**	0.000	0.000	0.000	0.000
YHR056C	**0.021**	0.014	0.014	0.014	**0.021**
YKL038W	0.000	**0.087**	0.000	0.000	0.000
YDR207C	0.010	**0.031**	0.000	0.000	0.000
YGL073W	**0.037**	0.015	0.021	0.012	0.028
YKL109W	0.052	**0.057**	0.031	0.022	0.044
YIR033W	0.000	**0.010**	0.000	0.000	0.000
YGL096W	**0.027**	0.004	0.016	0.020	**0.027**
YLR256W	**0.007**	0.000	0.000	0.000	0.000
YJR147W	**0.118**	**0.118**	**0.118**	**0.118**	**0.118**
YMR021C	0.000	**0.048**	0.000	0.000	0.000
YCL055W	**0.016**	**0.016**	**0.016**	**0.016**	**0.016**
YPL075W	**0.020**	**0.020**	**0.020**	**0.020**	**0.020**
YDR421W	0.032	**0.262**	0.032	0.016	0.016
YER161C	**0.024**	0.004	0.016	0.004	0.012
YBR083W	**0.031**	0.016	0.016	0.016	**0.031**
YIR017C	0.054	**0.071**	0.018	0.018	0.054
YFL031W	**0.059**	0.030	0.045	0.045	0.045
YLR098C	**0.012**	**0.012**	**0.012**	0.000	**0.012**
YNL216W	**0.013**	0.000	**0.013**	0.000	**0.013**
YDR216W	0.011	**0.044**	0.000	0.011	0.011
YDR096W	**0.100**	0.000	0.000	0.000	**0.100**
YER169W	**0.118**	0.000	**0.118**	**0.118**	**0.118**

TF NAME	PANAMA	LINEAR	SVA	ICE	PEER
YOR162C	**0.017**	0.000	0.000	0.000	0.000
YDR463W	**0.007**	0.000	0.000	0.000	**0.007**
YGL162W	**0.023**	0.008	0.008	0.000	**0.023**
YIL154C	**0.009**	0.000	**0.009**	0.000	**0.009**
YKL112W	**0.022**	0.018	0.018	0.013	**0.022**
YKL062W	**0.034**	**0.034**	**0.034**	**0.034**	**0.034**
YIL101C	**0.024**	0.000	0.012	0.000	**0.024**
YDR123C	0.012	0.000	**0.024**	0.000	0.012
YGL035C	**0.052**	0.038	0.031	0.024	0.031
YPR199C	0.057	**0.084**	0.017	0.017	0.057
YPL089C	0.000	**0.053**	0.000	0.000	0.000
YHR200W	0.070	**0.286**	0.000	0.000	0.033
YIL131C	**0.013**	0.000	0.000	0.000	**0.013**
YDL056W	0.018	0.004	**0.024**	0.015	0.018
YDL106C	0.005	0.000	**0.009**	0.000	0.000
YPL139C	**0.045**	**0.045**	0.034	0.034	**0.045**
YOL108C	0.088	**0.196**	0.000	0.018	0.088
YLR266C	**0.009**	**0.009**	**0.009**	**0.009**	**0.009**
YLR375W	0.012	**0.035**	0.000	0.000	0.012
YDR081C	**0.017**	0.011	0.011	0.011	**0.017**
YJR127C	0.022	**0.077**	0.022	0.015	0.022
YJL089W	**0.037**	0.025	0.025	0.012	**0.037**
YOR038C	**0.032**	0.005	0.016	0.005	**0.032**
YNL199C	**0.012**	0.006	0.010	0.006	**0.012**
YMR037C	**0.026**	0.013	0.022	0.013	0.020
YNL027W	0.000	**0.040**	0.000	0.000	0.000
YBL021C	**0.038**	**0.038**	**0.038**	**0.038**	**0.038**
YDR277C	**0.027**	0.013	**0.027**	0.020	**0.027**
YLR176C	**0.044**	0.022	0.000	0.022	**0.044**
YBR049C	**0.043**	0.022	0.000	0.022	0.011
YBL005W	0.017	**0.026**	0.017	0.009	0.017
