# Supplementary material for: Joint Modelling of Confounding Factors and Prominent Genetic Regulators Provides Increased Accuracy in Genetical Genomics Studies
Source: PLoS Comput Biol. 2012 Jan 5;8(1):e1002330. doi: 10.1371/journal.pcbi.1002330 (PMC3252274; doi:10.1371/journal.pcbi.1002330)
Supplement: Table S2 — F-score () for alternative methods in recovering known regulatory mechanisms from Yeastract. (PDF) [file pcbi.1002330.s013.pdf]

	Low rank	LMM	Preserves genetic signal
SVA	✓		✓(partially)
PEER	✓		✓(partially)
ICE		✓	
LMM-EH		✓	
**PANAMA**	✓	✓	✓
LINEAR			✓
