# Supplementary material for: Joint Modelling of Confounding Factors and Prominent Genetic Regulators Provides Increased Accuracy in Genetical Genomics Studies
Source: PLoS Comput Biol. 2012 Jan 5;8(1):e1002330. doi: 10.1371/journal.pcbi.1002330 (PMC3252274; doi:10.1371/journal.pcbi.1002330)
Supplement: Text S1 — Supplementary methods. (PDF) [file pcbi.1002330.s014.pdf]

Joint modelling of confounding factors and prominent genetic regulators provides increased accuracy in genetical genomics studies

Nicoló Fusi[1,†,*], Oliver Stegle[2,†,*], Neil D. Lawrence[1,*]
1 Sheffield Institute for Translational Neuroscience, University of Sheffield, UK
2 Machine Learning & Computational Biology Research Group, Max Planck Institute for Developmental Biology Tübingen, Germany
$*$ E-mail: nicolo.fusi@sheffield.ac.uk, oliver.stegle@tuebingen.mpg.de, N.Lawrence@sheffield.ac.uk
† these authors contributed equally

1 Statistical model of PANAMA

Here, we provide implementation details of the statistical model of PANAMA. A software implementation of PANAMA is freely available online at `http://ml.sheffield.ac.uk/qtl/`.

1.1 Derivation of a mixed linear model formulation

As outlined in Material and Methods, the statistical model underlying PANAMA assumes that the expression profiles are regulated by additive contributions from genetic factors as well as confounding factors. For a total of K observed SNPs $\mathbf{S} = (\mathbf{s}_1, \dots, \mathbf{s}_K)$ and effects from a dictionary of Q hidden factors $\mathbf{X} = (\mathbf{x}_1, \dots, \mathbf{x}_Q)$, the resulting generative model for G gene expression levels $\mathbf{Y} = (\mathbf{y}_1, \dots, \mathbf{y}_G)$ can be cast as

$$\mathbf{Y} = \boldsymbol{\mu} + \mathbf{SV} + \mathbf{XW} + \boldsymbol{\epsilon}. \tag{1}$$

We assume that expression levels and SNPs are observed in each of $n = 1, \dots, N$ individuals, $\boldsymbol{\mu} = (\mu_1, \dots, \mu_G)$ is a gene-specific mean effect and $\boldsymbol{\epsilon}$ denotes Gaussian distributed observation noise, $\epsilon_{n,g} \sim \mathcal{N}(0, \sigma_e^2)$. The matrix $\mathbf{V}$ represents the weight matrix for SNP effects and is of dimensionality SNPs $\times$ genes. Similarly, $\mathbf{W}$ denotes the weights of latent factors with dimensions genes $\times$ factors.

Parameter inference in the model implied by Equation (1) is difficult for common eQTL datasets, with the number of observed SNPs (K) and genes (G) greatly exceeding the number of samples (N). To address this regime of small sample sizes, we treat the model above as mixed linear model, imposing a hierarchy for the factor weights $\mathbf{W}$ and weight parameters of SNPs $\mathbf{V}$. We choose independent Gaussian priors for the factors weights $\mathbf{w}_q$ and the weights of the regulating SNPs $\mathbf{v}_k$

$$p(\mathbf{W}) = \prod_{q=1}^{Q} \mathcal{N}\left(\mathbf{w}_q \,\middle|\, \mathbf{0}, \alpha_q^2 \mathbf{I}\right),$$
$$p(\mathbf{V}) = \prod_{k=1}^{K} \mathcal{N}\left(\mathbf{v}_k \,\middle|\, \mathbf{0}, \beta_k^2 \mathbf{I}\right).$$

The variance parameters for each factor α_q^2 and each SNP β_k^2 modulate the relevance of the corresponding regulatory variables. Now, integrating over the weights $\mathbf{W}$ and $\mathbf{V}$ yields the marginal likelihood that factorizes across genes

$$p(\mathbf{Y} \,|\, \mathbf{S}, \mathbf{X}, \boldsymbol{\Theta}) = \prod_{g=1}^{G} \mathcal{N}\left(\mathbf{y}_g \,\middle|\, \mathbf{0}, \underbrace{\sum_{k=1}^{K} \beta_k^2 \mathbf{s}_k \mathbf{s}_k^{\mathrm{T}} + \sum_{q=1}^{Q} \alpha_q^2 \mathbf{x}_q \mathbf{x}_q^{\mathrm{T}} + \sigma_e^2 \mathbf{I}}_{\boldsymbol{\Sigma}}\right). \tag{2}$$

For notational convenience we dropped the mean term $\boldsymbol{\mu}$ and we have defined $\boldsymbol{\Theta} = \{\{\beta_k^2\}, \{\alpha_q^2\}, \sigma_e^2\}$, the set of all hyperparameters of the model.

1.2 Accounting for known covariates

Additive effects of covariates that are known can be straightforwardly included in the covariance structure of PANAMA $\boldsymbol{\Sigma}$

$$p(\mathbf{Y} \,|\, \mathbf{X}, \boldsymbol{\Theta}) = \prod_{g=1}^{G} \mathcal{N}\left(\mathbf{y}_g \,\middle|\, \mathbf{0}, \underbrace{\sum_{k=1}^{K} \beta_k^2 \mathbf{s}_k \mathbf{s}_k^{\mathrm{T}} + \sum_{q=1}^{Q} \alpha_q^2 \mathbf{x}_q \mathbf{x}_q^{\mathrm{T}} + \gamma^2 \mathbf{K}_0 + \sigma_e^2 \mathbf{I}}_{\boldsymbol{\Sigma}}\right). \quad (3)$$

Here, we included $\mathbf{K}_0$, denoting the covariance induced by these additional covariates. For example to account for a vector of fixed covariates $\mathbf{c}$, a corresponding background covariance can be constructed as $\mathbf{K}_0 = \mathbf{c}\mathbf{c}^{\mathrm{T}}$. It is also possible to account for the pairwise population-genetic relatedness of the samples by setting $\mathbf{K}_0$ to a kindship matrix that has been estimated on the complete set of SNPs; see for example Kang et al. [1] and references therein. Also in Listgarten et al. [2], the authors consider the additive combination of multiple confounding covariance structures in eQTL studies which is related to what is proposed here.

1.3 Parameter learning

Parameter learning, i.e. determining the most probable state of the hyperparameters $\boldsymbol{\Theta}$ and the latent factors $\mathbf{X}$, can be carried out using a straightforward maximum likelihood approach (Equation (2))

$$\{\hat{\boldsymbol{\Theta}}, \hat{\mathbf{X}}\} = \underset{\boldsymbol{\Theta}, \mathbf{X}}{\operatorname{argmax}} \underbrace{\ln p(\mathbf{Y} \,|\, \mathbf{S}, \mathbf{X}, \boldsymbol{\Theta})}_{\text{LML}} \quad (4)$$

$$= -\frac{NG}{2} \ln 2\pi - \frac{G}{N} \ln|\boldsymbol{\Sigma}| - \frac{1}{2} \mathrm{tr}(\boldsymbol{\Sigma}^{-1} \mathbf{Y} \mathbf{Y}^{\top}), \quad (5)$$

where the covariance $\boldsymbol{\Sigma}$ implicitly depends on the model parameters $\mathbf{X}$ and $\boldsymbol{\Theta}$. Analytical expression for the gradients of the objective function with respect to particular a particular element of the parameter set θ_i can be determined in closed form

$$\frac{\partial \text{LML}}{\partial \theta_i} = \frac{\partial \text{LML}}{\partial \boldsymbol{\Sigma}} \frac{\partial \boldsymbol{\Sigma}}{\partial \theta_i} = \left(\boldsymbol{\Sigma}^{-1} \mathbf{Y} \mathbf{Y}^{\top} \boldsymbol{\Sigma}^{-1} - G \boldsymbol{\Sigma}^{-1}\right) \frac{\partial \boldsymbol{\Sigma}}{\partial \theta_i}, \quad (6)$$

where $\frac{\partial \boldsymbol{\Sigma}}{\partial \theta_i}$ is the matrix derivative of the covariance with respect to a particular parameter. The objective function and gradients can be used in combination with a gradient-based optimizer such as the limited memory BFGS algorithm (L-BFGS, see [3]). Complete details on parameter inference in Gaussian process models can be found elsewhere [4, 5].

1.4 Determining the latent dimensionality

In addition to the hyperparameters, the latent dimensionality of the latent space Q is an important implicit parameter of factor-models such as PANAMA. Choosing Q too large results in over-correction, with the model explaining away true genetic associations. In contrast, choosing too few hidden factors, leads to under-correction, where the full hidden variation is not accounted for, ultimately leading to reduced sensitivity.

In related work, several of approaches have been proposed to select an appropriate latent dimensionality. One approach is to consider the explained variance, choosing a user-defined cutoff that determines the fraction of variance explained away by factor components. Alternatively, in [6], the authors estimate the number of factors using a permutation procedure alongside with additional heuristics that yield the expected number of target genes of a true confounding factor. Also in [7], Minka suggests to employ Bayesian model comparison, evaluating the marginal likelihood of the observed data in the light of alternative models that correspond to different choices of the latent dimensionality.

Here, we employ automatic relevance detection (ARD) [8,9]. The principle underlying ARD is to avoid choosing a cutoff value for the number of factors explicitly and instead determine an effective dimension of the latent space while learning. ARD has previously been used to identify a suitable latent dimension for confounder models in [10]. In PANAMA, the variance explained by each hidden factor is controlled by the values of α_q^2, with small values corresponding to irrelevant factors and larger values to factors that explain significant amounts of variation.

In practice, we first obtain a coarse estimate of the latent dimension by using PCA, choosing a cutoff point Q for the number of latent factors when 95% of the total variance is explained. This approach yields an upper bound of the latent dimensionality, which we use a starting point in PANAMA. The learning procedure of PANAMA then determines the number of factors with non-zero relevances α_q^2 automatically while optimizing the marginal likelihood (Equation (2)). This approach is both computational efficient and avoids the need of user specified tuning parameters.

The state of the latent factors is initialized by using a perturbed PCA solution (as suggested in [5]). Empirically, this approach yields similar results than initialising the factor randomly, however greatly increases the rate of convergence.

1.5 Iterative learning of the complete model

The presentation so far neglects a strategy to identify regulatory SNPs to be accounted for in the covariance structure (Equation (2)). Accounting for the complete set in the covariance is computationally infeasible and difficult to identify statistically, because the number of relevance parameters α_k typically exceeds the number of samples. Here, we suggest an iterative procedure, where only key regulators that are essential to accurately estimate the hidden factors are included during learning. In each iteration we add the SNPs that are most overlapping with the span of the current latent dimensionality, as defined by a linear association test between all latent factors and SNPs. As a convergence criterion we use a qvalue [11] cutoff for statistical significance of the association scan between factors and SNPs. In the following, we refer to this cutoff as FDR addition cutoff. A summary of the final PANAMA algorithm is outlined in Algorithm 1.

We checked that the performance of PANAMA is not sensitive to the exact setting of the FDR addition cutoff value. Figure 1a shows the impact on the performance of PANAMA (AUC) when using alternative cutoff values that regulate the extend of *trans* regulators to be included in the model covariance structure. Reassuringly, PANAMA approached the performance of the ideal model for less stringent cutoffs corresponding to a greater number of regulators that were included during the learning process. We also checked the calibratedness of the test statistics of PANAMA. In general, less stringent cutoffs that lead to larger numbers of regulators to be included in the model did not impact the calibration of resulting q-value estimates (See Figure 1b). Hence, in practical applications the increased computational cost of determining the genetic weight parameters β_k^2 is the limiting factor when choosing less stringent FDR addition cutoffs values.

Input: Matrix $\mathbf{Y}$ of individuals $\times$ genes, matrix $\mathbf{S}$ of individuals $\times$ SNPs
Output: Final covariance structure $\mathbf{\Sigma}$

initialize $\mathcal{I} = \emptyset$;
Estimate initial latent dimensionality from PCA $Q = \mathrm{PCA}(\mathbf{Y}, 95\%)$;
$\mathbf{X} = \mathrm{PCA}(\mathbf{Y}, Q) + \mathcal{N}(0, 1)$;
$t = 1$;
Initialise genetic regulators empty $\mathcal{I}_t = \{\}$;
repeat update $\{\boldsymbol{\theta}_{\mathrm{K}}, \mathbf{X}\}$:
  $(\boldsymbol{\theta}^*_{\mathrm{K}}, \mathbf{X}^*) = \mathrm{argmax}_{\mathbf{X}, \boldsymbol{\theta}_{\mathrm{K}}}\, p(\mathbf{Y} \mid \mathbf{S}, \mathbf{X}, \boldsymbol{\theta}_{\mathrm{K}}, \mathcal{I}_t)$; `/* optimise covariance */`
  $k^*, q^* = \mathrm{argmax}_{k,q}\, \mathrm{LOD}_{k,q}(\mathbf{s}_k, \mathbf{x}_q)$; `/* scan factor-SNP associations */`
  if *LOD_{k^*,q^*} significant (qv <FDR addition cutoff)* **then**
    $\mathcal{I}_{t+1} = \mathcal{I}_t \cup \{k^*\}$; `/* add overlapping SNP to covariance */`
  end
  $t = t + 1$
until $\mathcal{I}_t = \mathcal{I}_{t+1}$;

Algorithm 1: Algorithm summary of the iterative learning in performed in PANAMA. SNPs that overlap with current estimate of the hidden factors ($\mathbf{X}$) are greedily included in the covariance structure until convergence is reached.

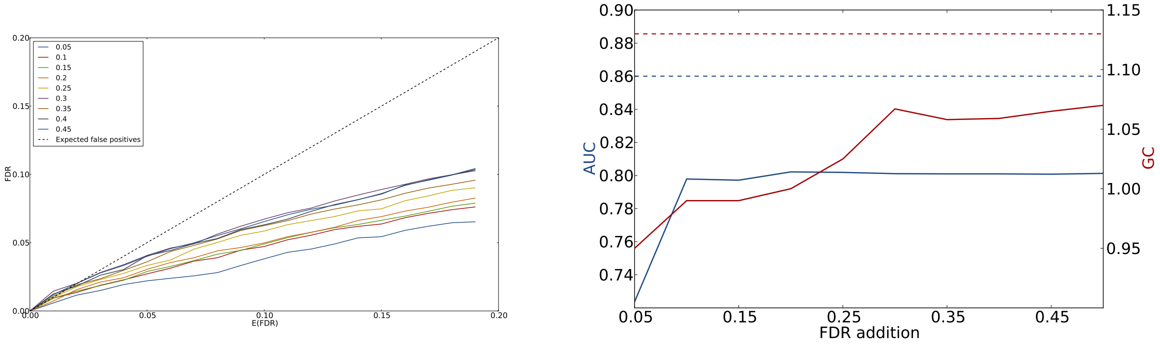

(a) E(FDR) vs FDR

(b) AUC/GC

Figure 1: Impact of choosing more stringent (0.05) to less stringent (0.5) cutoff parameters for adding *trans* associations into PANAMA while learning hidden confounders. **(a)** Estimated false discovery rate (E(FDR)) versus the empirical false discovery rate of called associations on the simulated dataset. **(b)** Area under the Receiver Operating Characteristics and inflation of the test statistics, λ. For comparison this figures includes AUC and λ of an ideal model, with the confounders being removed. The results show that PANAMA is not sensitive to the choice of the stringency parameter for including *trans* factors and generally achieves better performance for higher values. In the experiments reported in the main paper we used 0.5 throughout.

1.6 Testing strategies

Given a trained instance of the PANAMA model, the goal to use the inferred confounding factors to conduct statistical testing for individual eQTLs. Due to the formulation as variance component model, a linear mixed model (LMM) approach is a natural choice for association testing.

Mixed model testing approaches In an LMM, the trained covariance structure effectively acts as a random effect background model to account for non-genetic confounding variation. A wide range of statistical tests can be defined given the covariance structure; see for example [1, 12, 13] and references therein. Here, we employ a likelihood ratio test. For any pair of gene g and SNP k, this test statistics can be derived as

$$\mathrm{LOD}_{g,k} = \log \frac{\mathcal{N}\left(\mathbf{y}_g \mid \theta \mathbf{s}_k, \sigma_k^2 \mathbf{K} + \sigma_e^2 \mathbf{I}\right)}{\mathcal{N}\left(\mathbf{y}_g \mid \ \mathbf{0}\ \ , \sigma_k^2 \mathbf{K} + \sigma_e^2 \mathbf{I}\right)}, \tag{7}$$

where σ_k^2 and σ_e^2 weight the respective distribution of the confounding covariance $\mathbf{K}$ and additive noise contributions, which are refitted for every test. The confounding covariance matrix $\mathbf{K}$ is derived from components of the complete covariance $\mathbf{\Sigma}$ of the fitted PANAMA model (Equation (2)), with different choices corresponding to alternative correction strategies.

In standard PANAMA, the covariance $\mathbf{K}$ accounts for the confounding factors alone

$$\mathbf{K} = \sum_{q=1}^{Q} \alpha_q^2 \mathbf{x}_q \mathbf{x}_q^{\mathrm{T}}.$$

Alternatively, in PANAMA$_{\mathrm{trans}}$, also correcting for the *trans* factors, the covariance also includes *trans* regulators

$$\mathbf{K}_{\mathrm{trans}} = \sum_{k=1}^{K} \beta_k^2 \mathbf{s}_k \mathbf{s}_k^{\mathrm{T}} + \sum_{q=1}^{Q} \alpha_q^2 \mathbf{x}_q \mathbf{x}_q^{\mathrm{T}}.$$

As discussed in the main text, PANAMA$_{\mathrm{trans}}$ accounts for the putative confounding influence of broad variance components that do have a genetic basis. While these are not confounding *per se*, accounting for their effect may increase the power for identifying smaller effects that are otherwise overshadowed.

Efficient mixed model implementations Several computational advances have been presented to efficiently carry out the mixed model tests for all SNP/gene pairs (Equation (7)) [1,12,13]. In the software implementation that accompanies PANAMA, we follow the route taken in most recent development, allowing for exact inference while retaining linear-time complexity in the number of samples per test [12]. Similar as in EMMAX [1], we carry out a single cubical decomposition of the full-rank matrix $\mathbf{K}$ upfront. Briefly, the underlying idea is to decompose the testing covariance once, which allows to efficiently adapting the weights σ_e^2 and σ_k^2 for each individual test. These measures ensure PANAMA to be applicable to genome-scale datases (See Section 1.7).

PANAMA residuals for alternative downstream models For applications other than eQTL testing, it maybe desirable to account for the confounding factors explicitly, subtracting their contribution from the expression data. Such an approach is useful when using the expression levels in combination with other analyses such as clustering or network reconstruction.

In PANAMA, a residual dataset can be obtained by considering the joint Gaussian distribution on the observed data and the test dataset. Completing the square yields a closed form mean-prediction of this Gaussian covariance model

$$\hat{\mathbf{y}}_g = \mathbf{K}\left(\sigma_k^2 \mathbf{K} + \sigma_e^2 \mathbf{I}\right)^{-1} \mathbf{y}_g. \tag{8}$$

Similar as for mixed model testing, the relative weights of the correction and the noise component σ_k^2 and σ_e^2 are refit for every gene. See also [4] for further details on the usage of Gaussian models as predictors.

1.7 Software implementation and scalability

Due to the continuous increase in the size of genetically genomics studies, the computational efficiency of the current approaches for eQTL testing is of ever-increasing importance. The python implementation exploits several properties of the model, in order to allow for applicability to larger datasets. First, the marginal likelihood for parameter inference (Equation (4)) has a low-rank structure and hence allows for efficient evaluation of the matrix inverses, speeding up parameter learning (Section 1.3). Second, the association tests given the trained PANAMA model builds on recent advances for mixed models that scale linearly with the number of samples and tests [12].

Efficient testing and parallelization Typically, in large scale data the bottleneck lies in the association testing, thus demanding for particular attention of this step. PANAMA builds on recent advances for fast mixed model testing [12], which accompany the PANAMA software package in form of an integrated C++ library. While good performance on a single process/thread is needed, scientific software also requires to be easily parallelized for computing on clusters and clouds. To this end, PANAMA natively allows for jobs to be distributed across multiple processes, multiple machines on the local network, on a cluster and on the most popular cloud computing platforms (provided they have a working Python/numpy/scipy installation).

Empirical computational cost and runtime To compare the computational demands of PANAMA and alternative methods, we carried out a timing experiment on a benchmark dataset consisting of 193 samples, 8,598 genes and 8,311 SNPs (based on the cortical dataset, chromosome 17, as described in the main text). The size of this problem was chosen as to ensure that the slowest approach converges within an acceptable time interval. Table 1 shows the cpu-time required for each of the methods that were considered in the main manuscript. [1] All tests were performed on a GNU/Linux machine with an Intel(R) Xeon(R) X7542 CPU and 64 gigabytes of RAM, the python scientific libraries (Numpy and Scipy) were compiled against the Intel(R) Math Kernel Library.

We also extrapolated the computational runtime for current human-scale data, assuming 193 samples, 40,000 genes and 10 million SNPs. These estimates are based on the assumption that the final testing step dominates the computational cost in all methods. This is especially true for the methods that use a low-rank representation of the confounding factors (PANAMA, SVA, PEER), since their computational cost for learning of confounders scales with respect to the number of individuals, not with respect of the number of genes. PANAMA, carrying out iterative learning to derive the confounding covariance (Section 1.5) requires additional tests between the learnt factors and all SNPs (Algorithm 1). Importantly, because the typical number of confounders is much smaller than genes, this cost can be neglected in practice. Even with 10 million SNPs and 40 factors (more than the typical number of factors in human), this association scan only takes 3 hours compared to 137 days of computation that are needed for genome-wide application of mixed model tests between all SNPs and genes.

Model	CPU-time (in **minutes**)	projected CPU-time on human-scale dataset (in **days**)
LINEAR	35	136
SVA	39	150
PEER	45	152
PANAMA	62	159
ICE	8,540	33,197

[1] The computationally dominating testing step in LINEAR, SVA, PEER has been identically implemented in python; testing of PANAMA in C++ and ICE is fully based on R scripts from the authors. Such difference in the implementation may have implications for the exact runtime estimates provided.

Table 1: Empirical computation time for experiments on parts of the human cortical dataset (chromosome 17) and extrapolations for a full-genome dataset with 10 million SNPs and 40,000 probes.

2 Significance testing and multiple testing correction

In experiments, all considered methods were applied to carry out independent association test between individual SNPs and genes. We assessed genome-wide significance of individual associations using Storey's q-value method [11].

3 Synthetic dataset

The artificial dataset was created, mimicking key characteristics of the real yeast eQTL dataset (Yeast dataset, main text).

Simulation approach We first fit PANAMA to the real eQTL data, estimating the confounding variation and *cis* and *trans* associations. Given the fitted model of independent tests, we reduced the association matrix between all SNPs and genes to at most one association per chromosome and gene, avoiding inflated association counts due to linkage disequilibrium. To also include weak associations, we considered association with a q-value of at most 0.3. On the residual dataset, after removing the effect of the estimated confounders, we then fitted a linear model of all significant associations for each gene. Next, we estimated final residuals by removing the confounders and the fitted associations to estimate a distribution of noise levels across genes.

Next, we used the fitted model parameters from the real dataset to create a synthetic eQTL dataset with known ground truth associations. We considered the same number of simulated *cis* and *trans* associations as found on the real data as well as the empirical distribution of associations weights and noise estimates obtained form the empirical fit. Using the real genotypes we randomly chose associations between SNPs and genes, simulating effects drawing from the empirical distribution of weights. Finally, we added confounding variation by drawing a sample from the fitted confounding covariance structure and added simulated noise from the fitted distribution of noise levels.

Variation of fitted simulation parameters Comparative evaluation of methods on the simulated data were repeated for variations of the fitted simulation parameters (main text). To create datasets of variable levels of difficulty, we considered different numbers of true simulated *trans* regulators (Figure 2e) and different numbers of simulated confounders (Figure 2f). In both cases, we ran the same simulation approach as previously described, however removing random fractions of the simulated *trans* regulators or confounders respectively.

Alternative simulation using ICE for real data fitting The simulation procedure described yields eQTL datasets that share key properties with the real dataset used for fitting. For comparison, we repeated the fitting process using ICE as an alternative method to correct for confounders (main text). All other details on the exact simulation procedure remained identical. Results on the simulation study can be found in Supporting Figure2.

References

1. Kang H, Sul J, et al. (2010) Variance component model to account for sample structure in genome-wide association studies. Nat Genet 42: 348–354.

2. Listgarten J, Kadie C, Schadt E, Heckerman D (2010) Correction for hidden confounders in the genetic analysis of gene expression. Proc Natl Acad Sci U S A 107: 16465.

3. Byrd R, Lu P, Nocedal J, Zhu C (1995) A limited memory algorithm for bound constrained optimization. SIAM J Sci Comput 16: 1190–1208.

4. Rasmussen CE, Williams CKI (2006) Gaussian Processes for Machine Learning. MIT Press.

5. Lawrence N (2005) Probabilistic Non-linear Principal Component Analysis with Gaussian Process Latent Variable Models. J Mach Learn Res 6: 1783–1816.

6. Leek JT, Storey JD (2007) Capturing heterogeneity in gene expression studies by surrogate variable analysis. PLoS Genet 3: 1724–35.

7. Minka TP (2001) Automatic choice of dimensionality for PCA. Adv Neural Inf Process Syst : 598–604.

8. Mackay DJC (1995) Probable networks and plausible predictions - a review of practical Bayesian methods for supervised neural networks. Network: Computation in Neural Systems 6: 469–505.

9. Neal RM (1996) Bayesian Learning for Neural Networks. Springer.

10. Stegle O, Parts L, Durbin R, Winn J (2010) A Bayesian Framework to Account for Complex Non-Genetic Factors in Gene Expression Levels Greatly Increases Power in eQTL Studies. PLoS Comput Biol 6: e1000770.

11. Storey J, Tibshirani R (2003) Statistical significance for genomewide studies. Proc Natl Acad Sci U S A 100: 9440.

12. Lippert C, Listgarten J, Liu Y, Kadie C, Davidson R, et al. (2011) Fast linear mixed models for genome-wide association studies. Nat Methods 8: 833–835.

13. Kang H, Zaitlen N, Wade C, Kirby A, Heckerman D, et al. (2008) Efficient control of population structure in model organism association mapping. Genetics 178: 1709.
